# Uveitis as a Risk Factor for Developing Acute Myocardial Infarction in Ankylosing Spondylitis: A National Population-Based Longitudinal Cohort Study

**DOI:** 10.3389/fimmu.2021.811664

**Published:** 2022-01-11

**Authors:** Yi-Fen Lai, Ting-Yi Lin, Wu-Chien Chien, Chien-An Sun, Chi-Hsiang Chung, Yi-Hao Chen, Jiann-Torng Chen, Ching-Long Chen

**Affiliations:** ^1^Department of Ophthalmology, Tri-Service General Hospital, National Defense Medical Center, Taipei, Taiwan; ^2^Department of Medical Research, Tri-Service General Hospital, National Defense Medical Center, Taipei, Taiwan; ^3^School of Public Health, National Defense Medical Center, Taipei, Taiwan; ^4^Graduate of Life Sciences, National Defense Medical Center, Taipei, Taiwan; ^5^Department of Public Health, College of Medicine, Fu-Jen Catholic University, New Taipei City, Taiwan; ^6^Big Data Research Center, College of Medicine, Fu-Jen Catholic University, New Taipei City, Taiwan; ^7^Taiwanese Injury Prevention and Safety Promotion Association, Taipei, Taiwan

**Keywords:** uveitis, acute myocardial infarction, ankylosing spondylitis, cardiovascular disease, epidemiology

## Abstract

**Background:**

Ankylosing spondylitis (AS) is a chronic inflammatory disease. Excess cardiovascular risks were well recognized in patients with AS and were attributed to prolonged systemic inflammation. Uveitis is one of the most common extra-articular symptoms of AS and is also considered an indicator of systemic inflammation. This study aimed to investigate whether uveitis was a risk factor for developing acute myocardial infarction (AMI) in patients with AS using the National Health Insurance Research Database (NHIRD).

**Methods:**

Data were collected from the NHIRD over a fifteen-year period. Variables were analyzed using the Pearson chi-square test and Fisher’s exact test. Risk factors for the occurrence of AMI were examined by calculating hazard ratio. Kaplan-Meier analysis was performed to compare the cumulative incidence of AMI in the uveitis and non-uveitis cohorts.

**Results:**

A total of 5905 patients with AS were enrolled, including 1181 patients with uveitis (20%) and 4724 patients without uveitis (80%). The Kaplan–Meier method with the log-rank test showed that the uveitis group had a significantly higher cumulative hazard for patients with AMI than the non-uveitis group (p < 0.001). The adjusted hazard ratio (aHR) of AMI was higher in the uveitis group than in the non-uveitis group (aHR = 1.653, *p* < 0.001). Stratified analysis revealed that patients with uveitis had an increased risk of developing AMI regardless of their sex (male/female aHR = 1.688/1.608, p < 0.001). Patients with uveitis in all age groups were independently associated with an increased risk of developing AMI compared to those without uveitis (20–39 years/40–59 years/≥ 60 years, aHR = 1.550, 1.579, 3.240, p < 0.001). Patients with uveitis had a higher probability of developing AMI regardless of comorbidities. Uveitis patients with comorbidities had a higher risk of developing AMI compared to uveitis patients without comorbidities.

**Conclusion:**

Uveitis is a significant risk factor for developing AMI in patients with AS. Physicians should be aware of the potential cardiovascular risk in AS patients with uveitis, especially simultaneously with other traditional risk factors of AMI. Further prospective studies are needed to elucidate the underlying mechanism between uveitis and AMI in patients with AS.

## Introduction

Ankylosis spondylitis (AS) is a chronic inflammatory disease that predominantly affects the spine, with a peak onset between the ages of 20 years and 40 years (1). More than 85% of these patients have a specific human leukocyte antigen, known as the HLA-B27 antigen (2). Patients may also experience extra-articular manifestations, such as uveitis, psoriasis, and inflammatory bowel disease. Vascular involvement manifests as aortitis, perioartitis, and aortic valve regurgitation (3). Coronary artery involvement is extremely rare in AS, which is serious and life-threatening when acute myocardial infarction (AMI) occurs (4). As for many systemic inflammatory diseases, AS with excess cardiovascular mortality and morbidity has been well recognized in previous studies (5, 6). The underlying mechanism of AMI may be attributed to the systemic inflammation associated with AS. Prolonged inflammation-induced vascular endothelial injury may result in atherosclerosis and thrombosis, leading to AMI (7).

Uveitis is one of the most common extra-articular symptoms of AS. The prevalence of uveitis in AS is approximately 20–30% (8). Currently, uveitis is believed to be not only an intraocular inflammation but also an indicator of systemic inflammation. Previous studies have demonstrated that the levels of pro-inflammatory cytokines increase in tears, aqueous humor, and serum in active disease (9–11). Uveitis might reflect the severity of systemic inflammation in AS, since inflammation may cause more cytokine cascades toward coronary vascular endothelial injuries, uveitis might be a risk factor for the development of AMI in AS. However, no study has proved this hypothesis.

As AMI is associated with life-threatening consequences (especially in young adults), it is important to know how AS and AMI are associated, including the risk factors of AMI in AS. However, most studies on this subject are case reports or case series, and the lack of a large sample size to investigate the association. Therefore, we used the National Health Insurance Research Database (NHIRD), which contains a comprehensive longitudinal medical record from 23 million people in Taiwan. This approach resulted in a large number and varying features of patients with AS compared to previous studies. This study aimed to investigate the factors that determine the incidence of AMI in AS. In addition, the study elucidated whether uveitis could be a risk factor for the development of AMI in AS. Our ultimate goal is to raise the awareness of physicians about AMI in AS patients with uveitis and provide information to all physicians for multidisciplinary care.

## Material and Methods

### Research Database

Beginning in 1995, the National Health Insurance (NHI) program was launched in Taiwan. More than 23 million Taiwanese and non-Taiwanese residents (approximately 99.9% of the total population) were included. The National Health Insurance Research Database (NHIRD) contains comprehensive medical records, including outpatients, inpatients, diagnoses, prescriptions, and procedures, which are registered by the International Classification of Diseases, Ninth Revision, Clinical Modification (ICD-9-CM) codes. The ICD-9-CM codes used in this study are listed in **Table S1**. These data eliminate personal identification and are available in an electronic format for clinicians to conduct statistical analysis.

### Study Participants

**Figure 1:**
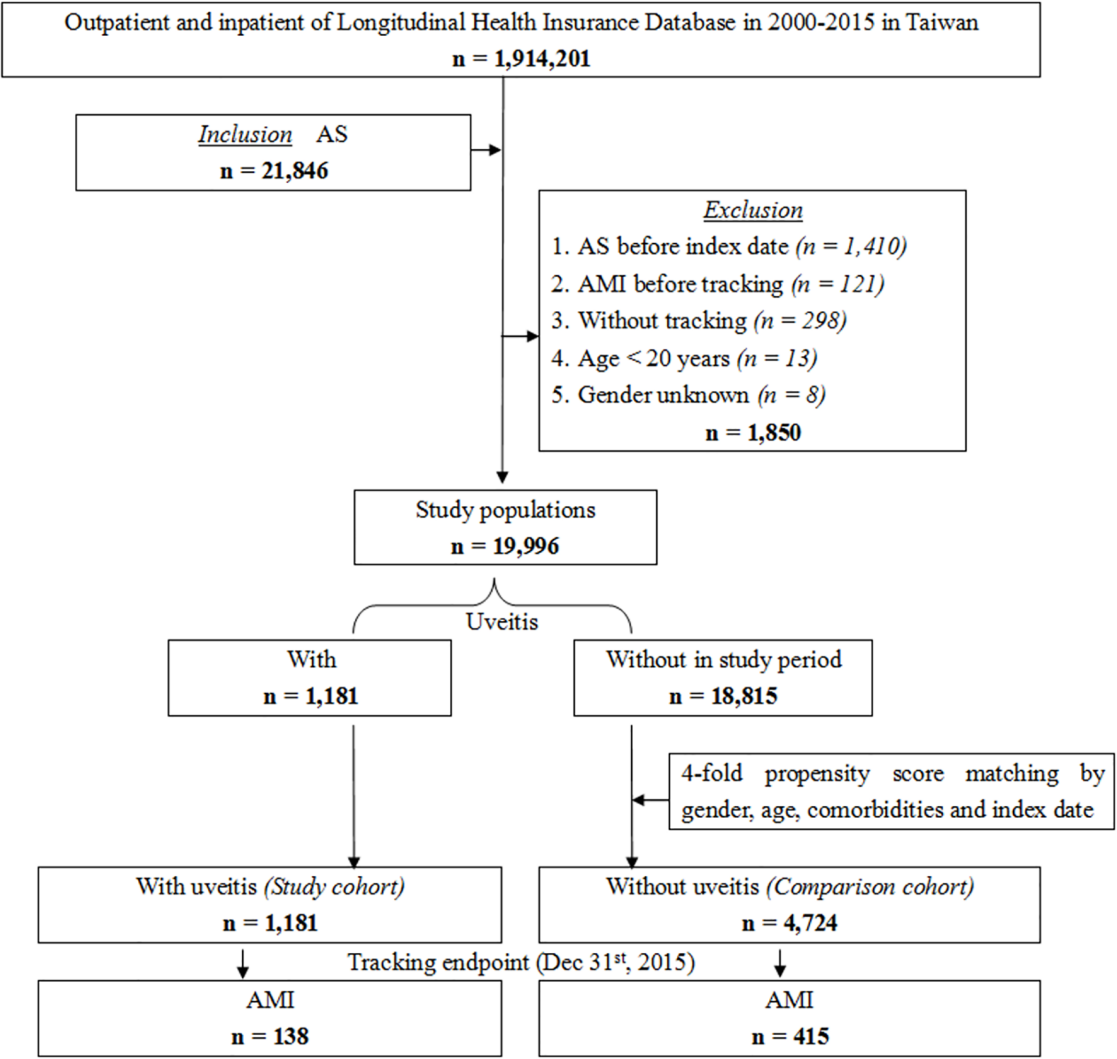
Flowchart of patient selection from the National Health Insurance Research Database in Taiwan.

We conducted a retrospective cohort study between January 2000 and December 2015. All included patients had at least one inpatient claim or outpatient visits more than three times with a diagnosis of AS according to the ICD-9-CM code (n = 21,846, ICD-9-CM code 720.0). Patients younger than 20 years old, with unknown sex, or without tracking were excluded. Patients diagnosed with AS and AMI before the index date were also excluded to ensure that patients had a new onset of AS and new onset of AMI after AS diagnosis. The index date was defined as the date of the first AS diagnosis. The study population was divided into the uveitis group (n = 1181, study cohort) and non-uveitic group (n = 18815). The group of non-uveitic patients matched four-fold with the uveitic group for sex, age, comorbidities, and index date as the comparison cohort (n = 4724). The uveitis group was divided into the anterior uveitis and posterior segment involvement groups. The populations in both groups did not overlap with each other. The uveitic subgroups and comorbidities extracted by ICD-9-CM codes are listed in **Table S1**. We replaced the Charlson comorbidity index (CCI) with the CCI_R (CCI excluding AMI, diabetes mellitus [DM], hyperlipidemia, hypertension [HTN], cerebrovascular accident [CVA], congestive heart failure [CHF], chronic obstructive pulmonary disease [COPD], asthma, coronary artery disease [CAD], cardiomegaly and metabolic syndrome [MetS]) because the removed diseases were also variables in this study. Patients were followed until AMI occurred (n = 138 in the uveitic group; n = 415 in the non-uveitic group) or until the end of the study period. **Figure 1** shows a flow chart of the patient selection process.

### Ethical Consideration

The study protocol was reviewed and approved by the institutional review board of the Tri-Service General Hospital (TSGH IRB No. B-110-35). Because the identities of all patients in the NHIRD were encrypted before data were released, the requirement for signed informed consent of the included patients was waived.

### Statistical Analysis

The Pearson chi-square test and Fisher’s exact test were used to evaluate differences in categorical variables such as sex, age group, and comorbidities. The threshold for statistical significance was set at p < 0.05. A Cox proportional hazard model was used to estimate the hazard ratio (HR) for the occurrence of AMI based on each variable in the univariate and multivariate analyses. Considering competing risk, Cox regression with Fine and Gray’s competing risk model was also employed for analysis. Kaplan–Meier analysis was performed to estimate the cumulative incidence of AMI in these two cohorts. All statistical analyses were performed using SPSS software (Version 22.0; SPSS Inc., Chicago, IL, USA).

## Results

**Table 1** shows the demographic and clinical characteristics of the enrolled patients with AS at baseline. A total of 5905 patients were enrolled, including 1181 (20%) patients with uveitis (uveitis group) and 4724 (80%) patients without uveitis (non-uveitis group). The non-uveitis group was matched four-fold with the uveitis group with age, sex, comorbidities, and index date. The mean age of the total AS patient was 37.72 ± 18.90 years, with the majority of patients (59.10%) younger than 40 years of age. Men accounted for 55.12% of the study population. For comparison of comorbidities at baseline, the prevalence of diabetes and hypertension was 25.15% and 20.51%, respectively. Those with chronic obstructive pulmonary disease and asthma accounted for 16.27% and 19.14% of the total AS cohort, respectively. Fewer patients (< 7%) had hyperlipidemia, cerebrovascular accident, congestive heart failure, coronary artery disease, cardiomegaly, and metabolic syndrome. At the end of follow-up, the percentages of AMI in the uveitis and non-uveitis groups were 11.69% and 8.78%, respectively (p < 0.001). All-cause mortality, which stands for death caused by any event, was 8.55% and 7.79% in the uveitis and non-uveitis groups, respectively (p = 0.332). The mortality caused by AMI in the uveitis group and non-uveitis group was 0.93% and 0.83%, respectively (p=0.723). The mean follow-up time is 9.91 ± 8.56 (range 0.01–15.99) years for all patients. The average time of AMI occurrence after patients included in the study was 2.90 ± 3.17 (range 0.03–15.82) years in the uveitis group and 3.64 ± 4.20 years (range 0.03–15.88) years in the non-uveitis group (p<0.001).

**Table 1:** Patients’ clinical characteristics at baseline.

Uveitis	Total		With		Without		*P*
Variables	n	%	n	%	n	%	
**Total**	5,905		1,181	20.00	4,724	80.00	
**Gender**							0.999
Male	3,255	55.12	651	55.12	2,604	55.12	
Female	2,650	44.88	530	44.88	2,120	44.88	
**Age (years) (mean ± SD)**	37.72 ± 18.90		37.61 ± 18.79		37.75 ± 18.97		0.868
**Age group (yrs)**							0.999
20-39	3,490	59.10	698	59.10	2,792	59.10	
40-59	1,655	28.03	331	28.03	1,324	28.03	
≧60	760	12.87	152	12.87	608	12.87	
**DM**							0.925
Without	4,420	74.85	883	74.77	3,537	74.87	
With	1,485	25.15	298	25.23	1,187	25.13	
**Hyperlipidemia**							0.810
Without	5,540	93.82	1,109	93.90	4,431	93.80	
With	365	6.18	72	6.10	293	6.20	
**HTN**							0.913
Without	4,694	79.49	940	79.59	3,754	79.47	
With	1,211	20.51	241	20.41	970	20.53	
**CVA**							0.825
Without	5,568	94.29	1,112	94.16	4,456	94.33	
With	337	5.71	69	5.84	268	5.67	
**CHF**							0.763
Without	5,730	97.04	1,145	96.95	4,585	97.06	
With	175	2.96	36	3.05	139	2.94	
**COPD**							0.881
Without	4,944	83.73	987	83.57	3,957	83.76	
With	961	16.27	194	16.43	767	16.24	
**Asthma**							0.835
Without	4,775	80.86	952	80.61	3,823	80.93	
With	1,130	19.14	229	19.39	901	19.07	
**CAD**							0.962
Without	5,545	93.90	1,108	93.82	4,437	93.92	
With	360	6.10	73	6.18	287	6.08	
**Cardiomegaly**							0.898
Without	5,845	98.98	1,170	99.07	4,675	98.96	
With	60	1.02	11	0.93	49	1.04	
**MetS**							0.880
Without	5,711	96.71	1,141	96.61	4,570	96.74	
With	194	3.29	40	3.39	154	3.26	
**CCI_R**	0.96 ± 1.11		0.99 ± 1.13		0.95 ± 1.11		0.162

DM, diabetes mellitus; HTN, hypertension; CVA, cerebrovascular accident; CHF, congestive heart failure; COPD, chronic obstructive pulmonary disease; CAD, coronary artery disease; MetS, metabolic syndrome; CCI_R, Charlson comorbidity index excluding acute myocardial infarction, diabetes mellitus, hyperlipidemia, hypertension, cerebrovascular accident, congestive heart failure, chronic obstructive pulmonary disease, asthma, coronary artery disease, cardiomegaly and metabolic syndrome.

**Figure 2:**
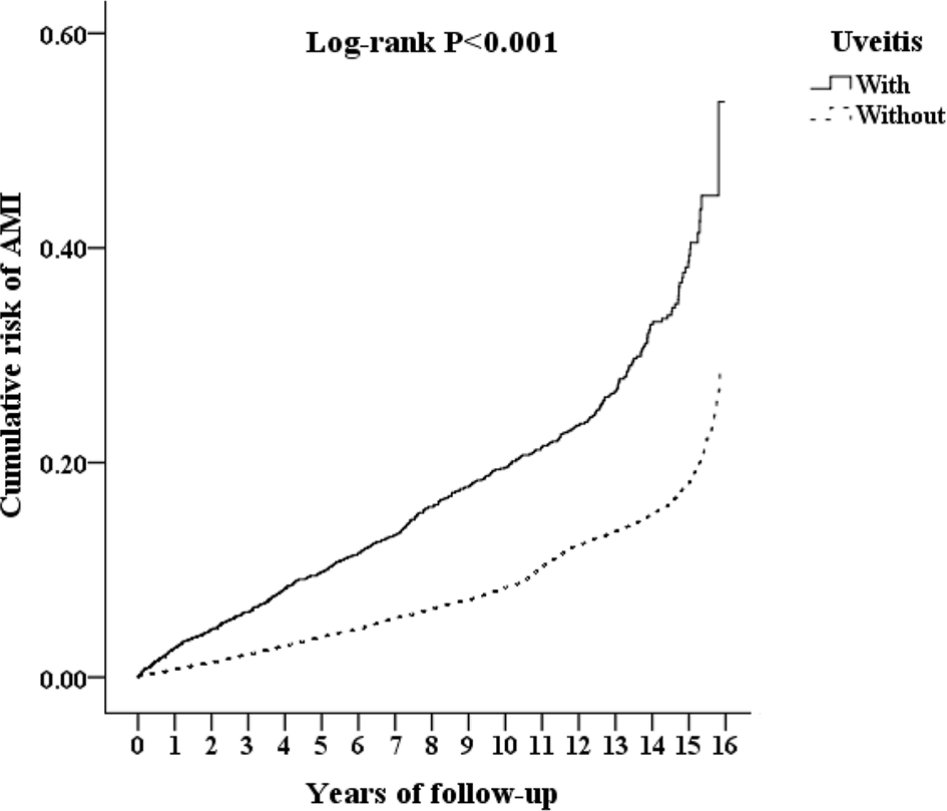
Kaplan-Meier analysis of the cumulative risk of AMI, stratified by uveitis with log-rank test. AMI, acute myocardial infarction.

The cumulative risk of developing AMI in patients with AS was calculated using the Kaplan–Meier method (**Figure 2**). The results showed that the uveitis group had a significantly higher hazard than the non-uveitis group (log-rank test, p < 0.001). The results of the risk factors for AMI using COX regression with and without Fine and Gray’s competing risk model are shown in **Table 2**. The adjusted HR (aHR) with competing risk analysis for AMI was 1.653 times greater in the uveitis group than in the non-uveitis group (95% CI 1.480–1.918). Although men were more likely to develop AMI, the result was not statistically significant after adjusting for confounders (aHR 1.179, p = 0.238). AS patients aged over 40 years showed a higher risk of developing AMI (aHR 1.11 in the 40–59 age group, p = 0.036; aHR 1.718 in the ≥ 60 age group, all p < 0.001). The hazard was significantly higher with age. Adjusted HRs showed that AS patients with DM, hyperlipidemia, HTN, CVA, CHF, COPD, asthma, CAD, and metabolic syndrome had a significantly higher risk of developing AMI, except for AS patients with cardiomegaly.

**Table 2:** Factors for AMI evaluated by Cox regression with/without Fine & Gray’s competing risk model.

Variables	No competing risk in the model								Competing risk in the model							
	Crude HR	95% CI		*P*	Adjusted HR	95% CI		*P*	Crude HR	95% CI		*P*	Adjusted HR	95% CI		*P*
**Uveitis**																
Without	Reference				Reference				Reference				Reference			
With	1.821	1.527	2.027	<0.001*	1.565	1.404	1.821	<0.001*	1.910	1.635	2.067	<0.001*	1.653	1.480	1.918	<0.001*
**Gender**																
Male	1.169	0.790	1.565	0.302	1.144	0.766	1.528	0.309	1.209	0.856	1.722	0.193	1.179	0.816	1.707	0.238
Female	Reference				Reference				Reference				Reference			
**Age (yrs)**																
20-39	Reference				Reference				Reference				Reference			
40-59	1.106	1.043	1.308	0.028*	1.074	1.003	1.266	0.047*	1.120	1.056	1.400	0.014*	1.111	1.016	1.347	0.036*
≧60	1.617	1.205	1.764	<0.001*	1.511	1.144	1.736	<0.001*	1.737	1.223	1.885	<0.001*	1.718	1.220	1.867	<0.001*
**DM**																
Without	Reference				Reference				Reference				Reference			
With	2.962	2.041	4.993	<0.001*	2.947	2.023	4.937	<0.001*	3.155	2.374	5.374	<0.001*	3.097	2.168	5.276	<0.001*
**Hyperlipidemia**																
Without	Reference				Reference				Reference				Reference			
With	2.714	1.560	4.312	<0.001*	2.586	1.508	4.203	<0.001*	2.954	1.917	4.991	<0.001*	2.948	1.817	4.906	<0.001*
**HTN**																
Without	Reference				Reference				Reference				Reference			
With	3.155	2.069	5.099	<0.001*	3.063	2.020	5.088	<0.001*	3.434	2.492	5.785	<0.001*	3.354	2.298	5.480	<0.001*
**CVA**																
Without	Reference				Reference				Reference				Reference			
With	2.225	1.366	2.734	<0.001*	2.137	1.244	2.652	<0.001*	2.284	1.407	2.828	<0.001*	2.226	1.368	2.736	<0.001*
**CHF**																
Without	Reference				Reference				Reference				Reference			
With	2.349	1.532	2.948	<0.001*	2.329	1.470	2.856	<0.001*	2.445	1.595	2.985	<0.001*	2.350	1.535	2.950	<0.001*
**COPD**																
Without	Reference				Reference				Reference				Reference			
With	1.479	1.054	2.448	0.017*	1.448	1.039	2.377	0.038*	1.551	1.102	2.596	<0.001*	1.514	1.068	2.518	0.002*
**Asthma**																
Without	Reference				Reference				Reference				Reference			
With	1.930	1.075	2.659	0.001*	1.919	1.041	2.606	0.009*	2.007	1.144	2.753	<0.001*	1.940	1.120	2.729	<0.001*
**CAD**																
Without	Reference				Reference				Reference				Reference			
With	4.188	2.289	7.094	<0.001*	4.090	2.191	7.017	<0.001*	4.334	2.399	7.263	<0.001*	4.172	2.224	7.033	<0.001*
**Cardiomegaly**																
Without	Reference				Reference				Reference				Reference			
With	0.000	-	-	0.999	0.000	-	-	0.999	0.000	-	-	0.999	0.000	-	-	0.999
**MetS**																
Without	Reference				Reference				Reference				Reference			
With	2.190	1.294	4.112	<0.001*	2.080	1.194	4.033	<0.001*	2.338	1.406	4.283	<0.001*	2.175	1.228	4.050	<0.001*
**CCI_R**	1.210	1.144	1.226	<0.001*	1.176	1.132	1.210	<0.001*	1.222	1.158	1.246	<0.001*	1.204	1.135	1.213	<0.001*

*Indicates statistical significance, p < 0.05. HR, hazard ratio; CI, confidence interval; Adjusted HR, adjusted variables are listed; DM, diabetes mellitus; HTN, hypertension; CVA, cerebrovascular accident; CHF, congestive heart failure; COPD, chronic obstructive pulmonary disease; CAD, coronary artery disease; MetS, metabolic syndrome; Competing risk, All-cause mortality; CCI_R, Charlson comorbidity index excluding acute myocardial infarction, diabetes mellitus, hyperlipidemia, hypertension, cerebrovascular accident, congestive heart failure, chronic obstructive pulmonary disease, asthma, coronary artery disease, cardiomegaly and metabolic syndrome.

In the stratified analysis comparing patients with and without uveitis (**Table 3**), the overall incidence of AMI was 1306.54 per 100,000 person-years in the uveitis group and 885.53 per 100,000 person-years in the non-uveitis group. Patients with uveitis have an increased risk of developing AMI regardless of their sex. The aHR was 1.688 in men and 1.608 in women (p < 0.001). Patients with uveitis in all age groups were independently associated with an increased risk of developing AMI than patients without uveitis, with an aHR of 1.550 in the 20–39 age group, 1.579 in the 40–59 age group, and 3.240 in the ≥ 60 age group (all p < 0.001). The aHR increased with age. For comorbidities, the uveitis group without DM, hyperlipidemia, HTN, CVA, CHF, COPD, asthma, CAD, cardiomegaly, and metabolic syndrome had an increased risk of developing AMI with aHRs of 1.550, 1.581, 1.439, 1.603, 1.659, 1.627, 1.597, 1.577, 1.650, and 1.615, respectively (all p < 0.001). The aHR was higher in the uveitis group with comorbidities than in the uveitis group without comorbidities. The uveitis group with DM, hyperlipidemia, HTN, CVA, CHF, COPD, asthma, CAD, and metabolic syndrome had increased aHR of developing AMI with aHRs of 3.287, 3.102, 4.166, 3.296, 3.261, 3.358, 3.286, 5.187, and 1.932, respectively (all p < 0.001).

**Table 3:** Factors for AMI stratified by variables listed in the table evaluated by Cox regression with/without Fine & Gray’s competing risk model.

Uveitis	With				Without *(Reference)*			No competing risk in the model				Competing risk in the model			
Stratified	Events		PYs	Rate (per 10^5^ PYs)	Events	PYs	Rate (per 10^5^ PYs)	Adjusted HR	95% CI		*P*	Adjusted HR	95% CI		*P*
**Total**	138	10,562.22		1,306.54	415	46,864.79	885.53	1.565	1.404	1.821	<0.001*	1.653	1.480	1.918	<0.001*
**Gender**															
Male	74	5,825.02		1,270.38	218	25,820.61	844.29	1.597	1.435	1.859	<0.001*	1.688	1.513	1.959	<0.001*
Female	64	4,737.20		1,351.01	197	21,044.18	936.13	1.522	1.366	1.771	<0.001*	1.608	1.442	1.867	<0.001*
**Age (yrs)**															
20-39	79	5,830.84		1,354.86	271	27,569.75	982.96	1.467	1.318	1.707	<0.001*	1.550	1.390	1.800	<0.001*
40-59	38	2,906.47		1,307.43	123	12,985.23	947.23	1.494	1.341	1.738	<0.001*	1.579	1.415	1.832	<0.001*
≧60	21	1,824.91		1,150.74	21	6,309.81	332.82	3.067	2.752	3.567	<0.001*	3.240	2.904	3.761	<0.001*
**DM**															
Without	109	7,902.89		1,379.24	388	38,557.65	1,006.29	1.468	1.318	1.708	<0.001*	1.550	1.391	1.801	<0.001*
With	29	2,659.33		1,090.50	27	8,307.14	325.02	3.110	2.792	3.619	<0.001*	3.287	2.947	3.815	<0.001*
**Hyperlipidemia**															
Without	124	9,912.46		1,250.95	400	44,890.56	891.06	1.497	1.344	1.742	<0.001*	1.581	1.417	1.836	<0.001*
With	14	649.76		2,154.64	15	1,974.23	759.79	2.936	2.635	3.416	<0.001*	3.102	2.781	3.602	<0.001*
**HTN**															
Without	103	8,455.25		1,218.18	386	39,762.35	970.77	1.361	1.222	1.585	<0.001*	1.439	1.289	1.669	<0.001*
With	35	2,106.97		1,661.15	29	7,102.44	408.31	3.942	3.540	4.587	<0.001*	4.166	3.735	4.837	<0.001*
**CVA**															
Without	130	9,963.59		1,304.75	403	44,382.68	908.01	1.517	1.362	1.766	<0.001*	1.603	1.438	1.861	<0.001*
With	8	598.63		1,336.38	12	2,482.11	483.46	3.119	2.800	3.629	<0.001*	3.296	2.955	3.826	<0.001*
**CHF**															
Without	136	10,257.13		1,325.91	413	46,214.91	893.65	1.571	1.410	1.827	<0.001*	1.659	1.487	1.927	<0.001*
With	2	305.09		655.54	2	649.88	307.75	3.085	2.771	3.590	<0.001*	3.261	2.923	3.785	<0.001*
**COPD**															
Without	128	8,843.30		1,447.42	402	40,662.92	988.62	1.540	1.383	1.792	<0.001*	1.627	1.459	1.889	<0.001*
With	10	1,718.92		581.76	13	6,201.87	209.61	3.177	2.852	3.697	<0.001*	3.358	3.010	3.898	<0.001*
**Asthma**															
Without	124	8,558.37		1,448.87	399	39,083.50	1,020.89	1.512	1.357	1.760	<0.001*	1.597	1.431	1.855	<0.001*
With	14	2,003.85		698.66	16	7,781.29	205.62	3.110	2.791	3.619	<0.001*	3.286	2.946	3.815	<0.001*
**CAD**															
Without	129	9,929.33		1,299.18	407	44,254.64	919.68	1.492	1.340	1.737	<0.001*	1.577	1.414	1.831	<0.001*
With	9	632.89		1,422.05	8	2,610.15	306.50	4.909	4.407	5.712	<0.001*	5.187	4.649	6.022	<0.001*
**Cardiomegaly**															
Without	138	10,446.59		1,321.01	415	46,685.97	888.92	1.562	1.402	1.817	<0.001*	1.650	1.479	1.916	<0.001*
With	0	115.63		0.00	0	178.82	0.00	–	–	–	-	–	–	–	-
**MetS**															
Without	127	10,324.48		1,230.09	409	46,566.68	878.31	1.492	1.277	1.741	<0.001*	1.615	1.392	1.764	<0.001*
With	11	237.74		4,626.90	6	298.11	2,012.68	1.744	1.451	2.209	<0.001*	1.932	1.587	2.436	<0.001*

*Indicates statistical significance, p < 0.05. PYs, Person-years; Adjusted HR, Adjusted Hazard ratio: adjusted for the variables listed in **Table 2**; CI, confidence interval; Competing risk: all-cause mortality.

The uveitis group (n = 1181) was further divided into the anterior uveitis group (36.6%, n = 432) and posterior segment involvement group (63.4%, n = 749) compared to the non-uveitis group (n = 4724) (**Table 4**). The overall incidence of AMI was 885.53 per 100,000 person-years in the non-uveitis group, 1,211.31 per 100,000 person-years in the anterior uveitis group, and 1,371.76 person-years in the posterior segment involvement group. The aHR was 1.624 in the anterior uveitis group and 1.673 in the posterior segment involvement group. Uveitis patients with posterior segment involvement had a slightly increased aHR for developing AMI than patients with only anterior uveitis.

**Table 4:** Factors for AMI among different uveitis subgroup evaluated by Cox regression with/without Fine & Gray’s competing risk model.

					No competing risk in the model				Competing risk in the model			
Uveitis subgroup	Populations	Events	PYs	Rate (per 10^5^ PYs)	Adjusted HR	95% CI	95% CI	*P*	Adjusted HR	95% CI	95% CI	*P*
Without uveitis	4,724	415	46,864.79	885.53	Reference				Reference			
With uveitis	1,181	138	10,562.22	1,306.54	1.565	1.404	1.821	<0.001*	1.653	1.480	1.918	<0.001*
Anterior uveitis	432	52	4,292.89	1,211.31	1.541	1.394	1.779	<0.001*	1.624	1.409	1.874	<0.001*
Posterior segment involvement	749	86	6,269.33	1,371.76	1.595	1.436	1.848	<0.001*	1.673	1.510	1.930	<0.001*

*Indicates statistical significance, p < 0.05. PYs, Person-years; Adjusted HR, adjusted hazard ratio: adjusted for the variables listed in **Table 2**; CI, confidence interval; Competing risk: all-cause mortality.

## Discussion

In this population-based cohort study, we enrolled 5905 patients with newly diagnosed AS. Of these patients, 1181 had uveitis and 4724 did not have uveitis. In the mean follow-up of 9.91 years, we found that patients with uveitis had a significantly higher risk (aHR = 1.653) for developing AMI than patients without uveitis. Kaplan–Meier analysis also revealed that the cumulative risk of developing AMI was significantly increased in the uveitis group. In the stratified analysis, the incidence of AMI in the uveitis group was 1306.54 per 100,000 person-years and 1.47-fold higher than that in the non-uveitis group. Uveitis was associated with an increased risk of developing AMI in both sexes, all age groups, patients with and without comorbid DM, hyperlipidemia, HTN, CVA, CHF, COPD, asthma, CAD, and metabolic syndrome. Uveitis patients with comorbidities had a higher adjusted hazard of developing AMI compared to uveitis patients without comorbidities.

The prevalence of AS has geographical differences. AS is more common in Europe and Asia than in Latin America (12). Previous studies have shown that the prevalence of AS per 10000 is highest in Turkey (49.0), lower in Taiwan (33.7), India (7.0), and lowest in Mexico (2.6) (13–16). The male-to-female ratio in AS also varies across countries, ranging between 1.2 and 7.0 (13, 17); however, these results consistently showed a male predominance. In the present study, the male-to-female ratio was 1.23, which is similar to the results previous study in Asia (18). Previous studies have shown that AS is usually diagnosed in the second or fourth decade of life (19, 20). In our study, more than half of the participants (59.10%) was consistently between 20–40 years of age. In our report, 5.9% of patients with AS had uveitis (1181/19996 = 5.90%). The prevalence of uveitis in AS varies in different studies, ranging from 0% to 30% (8, 21–23) and was even lower (0%) in a Philippines hospital-based study (24).

For comorbidities, the percentages of hypertension and chronic obstructive pulmonary disease were similar to those reported in previous studies (25, 26). The percentage of patients with diabetes and asthma was slightly higher than those reported in previous studies. The percentage of diabetes in a study carried out in the US was reported to be 17.3% in whites and 27.2% in African Americans (27). Asthma patients accounted for 7.3% of a German cohort (28). Our study population comprised AS patients with uveitis and AS patients without uveitis matched by AS patients with uveitis. The difference in the percentage of comorbidities might be attributed to race and the study population. Because of matching, the difference in baseline characteristics between the uveitis and non-uveitis groups was not statistically significant (**Table 1**). Therefore, the higher cumulative risk of developing AMI in the uveitis group demonstrated by the Kaplan–Meier curve (**Figure 2**) is less likely to be attributed to the differences in sex, age, and baseline systemic disease.

**Table 2** shows the variables associated with the development of AMI by Cox regression. As expected, patients with traditional risk factors had a higher risk of developing AMI, a result that is similar to those of previous studies (29, 30). Bremander et al. reported elevated morbidity ratios for hypertension and diabetes in AS patient with ischemic heart disease (29) Chou et al. found that AS patients with hypertension, diabetes, hyperlipidemia and stroke had an aHR of 7.74 for acute coronary syndrome compared to those without these comorbidities (30).

Our report also identified uveitis as an independent risk factor for AMI in patients with AS (**Table 3**). The reason why uveitis might be a risk factor for developing AMI in patients with AS is not clear. Uveitis may be an indicator of disease activity and systemic inflammation in AS (9, 31, 32). Patients with acute anterior uveitis have higher disease activity and inflammatory cytokines in the serum (9, 33). Since inflammation plays an important role in myocardial infarction (7), uveitis may be associated with cardiovascular disease. A cross-sectional study reported that AS patients with a history of uveitis were more predisposed to atherosclerosis (34). Another study also reported that children with a longer duration of uveitis had an increased frequency of deteriorated vascular function and carotid artery intima-media thickness (35).

Previous studies have reported that uveitis in spondyloarthritis is associated with HLA-B27, possibly acting through the IL-23/IL-17 axis (36–38). The activation of Th17 cells with Th17-related proinflammatory cytokine induction (IFN-γ, TNF-α, IL-1β, IL-6, IL-23) could result in spondyloarthritis with associated uveitis (39). Several studies have reported an association between Th-17-related proinflammatory cytokines and atherosclerosis. A thin fibrous cap in the lesion, which is indicative of unstable plaques prone to rupture and increased atherosclerosis correlate with increased Th17 cells, IL-23-producing vascular muscle, and macrophages in apoE/IL18 double-KO mice (40). Circulating Th17-related cytokines (IL-17, IL-6, IL-23) increased at the onset of acute coronary syndrome (41). The blockade of IL-17A in mice resulted in a significant reduction in atherosclerosis (42, 43). Thus, the IL-23/IL-17 axis is a possible common pathway of origin in uveitis and AMI.

We further divided uveitic patients into the anterior uveitic and posterior segment involvement groups (**Table 4**). Our study showed that the posterior segment involvement group accounted for the majority (63.4%) of the uveitis cohort. Most studies focused on the association between anterior uveitis and AS. Few studies have discussed the manifestation of posterior segment involvement in patients with AS uveitis. The percentage of posterior segment involvement in HLA-B27 associated uveitis was reported to be 17.4–63%, varying from one study to another (44–47). This difference might be attributed to race, study design, and study year. Our study also showed that the posterior segment involvement group had slightly increased aHR of developing AMI than the anterior uveitis group; however, we did not perform statistical analysis to compare the two groups. Due to the lack of further statistical information, the interpretation of higher aHR in the posterior segment involvement group should be done carefully.

Our study has several strengths. First, the National Health Administration in Taiwan checks charts to ensure patient diagnosis with appropriate treatment. Treatment followed a standardized protocol. The diagnoses were verified. Second, we used a comprehensive, longitudinal, nationwide database spanning 15 years. The NHI system was introduced in Taiwan in 1995, so we could conduct a longitudinal analysis of sequential events. AS or AMI before the index date could be excluded to eliminate bias in the cross-sectional study. Third, the coverage rate in Taiwan was approximately 99%. A large sample provides high statistical power. Finally, the major strength was that the findings after adjusting for confounders were highly consistent, suggesting reliable and convincing results.

However, this study had some limitations. First, the study was based on a retrospective statistical analysis from a database. The NHIRD database does not provide laboratory data, such as the erythrocyte sedimentation rate or C-reactive protein, as advocatory evidence. There were no clinical images such as MRI findings for classification of disease activity and severity of AS patients, which might also show a correlation with uveitis. Second, some traditional factors for AMI, such as diet, smoking, and physical activity, were not in the database. Third, AS-uveitis complex is most frequently observed as anterior uveitis and responds well to topical corticosteroids, whereas posterior uveitis is usually refractory to topical treatments and frequently requires treatments with systemic corticosteroids and anti-TNF agents to reduce recurrences. Cardiovascular events are complications of corticosteroids. In this study, we did not assess the influence of uveitis treatments in developing AMI. Fourth, the rates of all extra-articular manifestation in AS patients might influence the development of AMI, but the database did not provide the information of all extra-articular manifestation. Fifth, the included patients in our study are homogenous ethnic population with specific genetic and disease features as indicated by the prevalence of co-morbidities, type of uveitis and probably age at diagnosis. Hence, the results of our study must be confirmed in other ethnic populations. Sixth, this study had a retrospective design. We could only elucidate a possible mechanism based on this association. The causality needs further prospective studies with detailed information to confirm our conclusion.

## Conclusion

Uveitis is a risk factor for developing AMI in patients with AS. Doctors should be aware of the potential cardiovascular risk in patients with AS uveitis, especially simultaneously with other traditional risk factors for AMI. Closely monitoring the cardiovascular system in these patients is important for all physicians to detect AMI earlier and manage it promptly. Further prospective studies are needed to elucidate the underlying mechanism between uveitis and AMI in patients with AS.

## Data Availability Statement

The original contributions presented in the study are included in the article/**Supplementary Material**. Further inquiries can be directed to the corresponding author.

## Ethics Statement

The studies involving human participants were reviewed and approved by The institutional review board of the Tri-Service General Hospital (TSGH IRB No. B-110-35). Written informed consent for participation was not required for this study in accordance with the national legislation and the institutional requirements.

## Author Contributions

Conceptualization: Y-FL, W-CC, and C-LC. Methodology: W-CC and C-HC. Software: W-CC, C-AS, and C-HC. Validation: T-YL, W-CC, and C-HC. Formal analysis: W-CC, C-AS, and C-HC. Investigation: T-YL, Y-HC, and J-TC. Resources: W-CC, Y-HC, J-TC, and C-LC. Data curation: W-CC, C-HC, and C-LC. Writing—original draft preparation: Y-FL. Writing—review and editing: Y-FL, T-YL, and C-LC. Visualization: W-CC and C-HC. Supervision: W-CC, Y-HC, and C-LC. Project administration, Y-HC and C-LC. Funding acquisition, W-CC, J-TC, and C-LC. All authors have read and agreed to the published version of the manuscript.

## Funding

This study was supported by the Tri-Service General Hospital Research Foundation(TSGH-D-110112, TSGH-D-110109) and by the Ministry of National Defense (MNDMAB-110–084, MAB-E-110001). The sponsor has no role in study design, data collection and analysis, decision to publish, or preparation of the manuscript.

## Conflict of Interest

The authors declare that the research was conducted in the absence of any commercial or financial relationships that could be construed as a potential conflict of interest.

## Publisher’s Note

All claims expressed in this article are solely those of the authors and do not necessarily represent those of their affiliated organizations, or those of the publisher, the editors and the reviewers. Any product that may be evaluated in this article, or claim that may be made by its manufacturer, is not guaranteed or endorsed by the publisher.

## References

[1] Feldtkeller E Khan MA van der Heijde D van der Linden S Braun J. Age at Disease Onset and Diagnosis Delay in HLA-B27 Negative vs. Positive Patients With Ankylosing Spondylitis. Rheumatol Int (2003) 23(2):61–6. doi: 10.1007/s00296-002-0237-4 12634937

[2] Luo F Zhao Z Zhang J Leng J. Comparison of HLA-B*27 Subtypes Between Chinese Patients With Ankylosing Spondylitis and Non-Ankylosing Spondylitis Carriers. J Int Med Res (2019) 47(7):3171–8. doi: 10.1177/0300060519853929 PMC6683902 31177886

[3] Prati C Demougeot C Guillot X Sondag M Verhoeven F Wendling D. Vascular Involvement in Axial Spondyloarthropathies. Joint Bone Spine (2019) 86(2):159–63. doi: 10.1016/j.jbspin.2018.05.003 29787813

[4] Mathieu S Pereira B Soubrier M. Cardiovascular Events in Ankylosing Spondylitis: An Updated Meta-Analysis. Semin Arthritis Rheumat (2015) 44(5):551–5. doi: 10.1016/j.semarthrit.2014.10.007 25455683

[5] Eriksson JK Jacobsson L Bengtsson K Askling J. Is Ankylosing Spondylitis a Risk Factor for Cardiovascular Disease, and How Do These Risks Compare With Those in Rheumatoid Arthritis? Ann Rheumat Dis (2017) 76(2):364–70. doi: 10.1136/annrheumdis-2016-209315 27283333

[6] Huang YP Wang YH Pan SL. Increased Risk of Ischemic Heart Disease in Young Patients With Newly Diagnosed Ankylosing Spondylitis–A Population-Based Longitudinal Follow-Up Study. PloS One (2013) 8(5):e64155. doi: 10.1371/journal.pone.0064155 23691161 PMC3655062

[7] Hansson GK. Inflammation, Atherosclerosis, and Coronary Artery Disease. N Engl J Med (2005) 352(16):1685–95. doi: 10.1056/NEJMra043430 15843671

[8] Zeboulon N Dougados M Gossec L. Prevalence and Characteristics of Uveitis in the Spondyloarthropathies: A Systematic Literature Review. Ann Rheumat Dis (2008) 67(7):955–9. doi: 10.1136/ard.2007.075754 17962239

[9] Jawad S Liu B Agron E Nussenblatt RB Sen HN. Elevated Serum Levels of Interleukin-17A in Uveitis Patients. Ocular Immunol Inflamm (2013) 21(6):434–9. doi: 10.3109/09273948.2013.815786 PMC5569243 23957503

[10] Curnow SJ Falciani F Durrani OM Cheung CM Ross EJ Wloka K. Multiplex Bead Immunoassay Analysis of Aqueous Humor Reveals Distinct Cytokine Profiles in Uveitis. Invest Ophthalmol Visual Sci (2005) 46(11):4251–9. doi: 10.1167/iovs.05-0444 16249505

[11] Carreño E Portero A Herreras JM García-Vázquez C Whitcup SM Stern ME. Cytokine and Chemokine Tear Levels in Patients With Uveitis. Acta Ophthalmol (2017) 95(5):e405–14. doi: 10.1111/aos.13292 27873479

[12] Dean LE Jones GT MacDonald AG Downham C Sturrock RD Macfarlane GJ. Global Prevalence of Ankylosing Spondylitis. Rheumatol (Ox Engl) (2014) 53(4):650–7. doi: 10.1093/rheumatology/ket387 24324212

[13] Onen F Akar S Birlik M Sari I Khan MA Gurler O. Prevalence of Ankylosing Spondylitis and Related Spondyloarthritides in an Urban Area of Izmir, Turkey. J Rheumatol (2008) 35(2):305–9. 18085733

[14] Chou CT Pei L Chang DM Lee CF Schumacher HR Liang MH. Prevalence of Rheumatic Diseases in Taiwan: A Population Study of Urban, Suburban, Rural Differences. J Rheumatol (1994) 21(2):302–6. 8182641

[15] Joshi VL Chopra A. Is There an Urban-Rural Divide? Population Surveys of Rheumatic Musculoskeletal Disorders in the Pune Region of India Using the COPCORD Bhigwan Model. J Rheumatol (2009) 36(3):614–22. doi: 10.3899/jrheum.080675 19208598

[16] Alvarez-Nemegyei J Peláez-Ballestas I Sanin LH Cardiel MH Ramirez-Angulo A Goycochea-Robles MV. Prevalence of Musculoskeletal Pain and Rheumatic Diseases in the Southeastern Region of Mexico. A COPCORD-Based Community Survey. J Rheumatol Suppl (2011) 86:21–5. doi: 10.3899/jrheum.100954 21196595

[17] De Angelis R Salaffi F Grassi W. Prevalence of Spondyloarthropathies in an Italian Population Sample: A Regional Community-Based Study. Scand J Rheumatol (2007) 36(1):14–21. doi: 10.1080/03009740600904243 17454930

[18] Davatchi F Jamshidi AR Banihashemi AT Gholami J Forouzanfar MH Akhlaghi M. WHO-ILAR COPCORD Study (Stage 1, Urban Study) in Iran. J Rheumatol (2008) 35(7):1384. 18464299

[19] Trontzas P Andrianakos A Miyakis S Pantelidou K Vafiadou E Garantziotou V. Seronegative Spondyloarthropathies in Greece: A Population-Based Study of Prevalence, Clinical Pattern, and Management. The ESORDIG Study. Clin Rheumatol (2005) 24(6):583–9. doi: 10.1007/s10067-005-1106-9 15864686

[20] Cakır N Pamuk ÖN Derviş E Imeryüz N Uslu H Benian Ö. The Prevalences of Some Rheumatic Diseases in Western Turkey: Havsa Study. Rheumatol Int (2012) 32(4):895–908. doi: 10.1007/s00296-010-1699-4 21229358

[21] Stolwijk C van Tubergen A Castillo-Ortiz JD Boonen A. Prevalence of Extra-Articular Manifestations in Patients With Ankylosing Spondylitis: A Systematic Review and Meta-Analysis. Ann Rheumat Dis (2015) 74(1):65–73. doi: 10.1136/annrheumdis-2013-203582 23999006

[22] Hsu YR Huang JC Tao Y Kaburaki T Lee CS Lin TC. Noninfectious Uveitis in the Asia-Pacific Region. Eye (Lond Engl) (2019) 33(1):66–77. doi: 10.1038/s41433-018-0223-z PMC6328561 30323327

[23] Chen SC Chuang CT Chu MY Sheu SJ. Patterns and Etiologies of Uveitis at a Tertiary Referral Center in Taiwan. Ocular Immunol Inflamm (2017) 25(sup1):S31–8. doi: 10.1080/09273948.2016.1189577 27463023

[24] Abaño JM Galvante PR Siopongco P Dans K Lopez J. Review of Epidemiology of Uveitis in Asia: Pattern of Uveitis in a Tertiary Hospital in the Philippines. Ocular Immunol Inflamm (2017) 25(sup1):S75–80. doi: 10.1080/09273948.2017.1335755 29083984

[25] Haroon NN Paterson JM Li P Inman RD Haroon N. Patients With Ankylosing Spondylitis Have Increased Cardiovascular and Cerebrovascular Mortality: A Population-Based Study. Ann Intern Med (2015) 163(6):409–16. doi: 10.7326/M14-2470 26258401

[26] Sharif K Watad A Tiosano S Yavne Y Blokh Kerpel A Comaneshter D. The Link Between COPD and Ankylosing Spondylitis: A Population Based Study. Eur J Intern Med (2018) 53:62–5. doi: 10.1016/j.ejim.2018.04.002 29631757

[27] Singh DK Magrey MN. Racial Differences in Clinical Features and Comorbidities in Ankylosing Spondylitis in the United States. J Rheumatol (2020) 47(6):835–8. doi: 10.3899/jrheum.181019 31474592

[28] Rudwaleit M Andermann B Alten R Sörensen H Listing J Zink A. Atopic Disorders in Ankylosing Spondylitis and Rheumatoid Arthritis. Ann Rheumat Dis (2002) 61(11):968–74. doi: 10.1136/ard.61.11.968 PMC1753933 12379517

[29] Bremander A Petersson IF Bergman S Englund M. Population-Based Estimates of Common Comorbidities and Cardiovascular Disease in Ankylosing Spondylitis. Arthritis Care Res (2011) 63(4):550–6. doi: 10.1002/acr.20408 21452267

[30] Chou CH Lin MC Peng CL Wu YC Sung FC Kao CH. A Nationwide Population-Based Retrospective Cohort Study: Increased Risk of Acute Coronary Syndrome in Patients With Ankylosing Spondylitis. Scand J Rheumatol (2014) 43(2):132–6. doi: 10.3109/03009742.2013.822097 24134400

[31] Chen CH Lin KC Chen HA Liao HT Liang TH Wang HP. Association of Acute Anterior Uveitis With Disease Activity, Functional Ability and Physical Mobility in Patients With Ankylosing Spondylitis: A Cross-Sectional Study of Chinese Patients in Taiwan. Clin Rheumatol (2007) 26(6):953–7. doi: 10.1007/s10067-006-0403-2 17021671

[32] Robertson LP Davis MJ. A Longitudinal Study of Disease Activity and Functional Status in a Hospital Cohort of Patients With Ankylosing Spondylitis. Rheumatol (Ox Engl) (2004) 43(12):1565–8. doi: 10.1093/rheumatology/keh386 15353608

[33] Przepiera-Będzak H Fischer K Brzosko M. Extra-Articular Symptoms in Constellation With Selected Serum Cytokines and Disease Activity in Spondyloarthritis. Mediators Inflamm (2016) 2016:7617954. doi: 10.1155/2016/7617954 28053373 PMC5174179

[34] Berg IJ Semb AG van der Heijde D Kvien TK Hisdal J Olsen IC. Uveitis is Associated With Hypertension and Atherosclerosis in Patients With Ankylosing Spondylitis: A Cross-Sectional Study. Semin Arthritis Rheumat (2014) 44(3):309–13. doi: 10.1016/j.semarthrit.2014.05.017 24968705

[35] Conkar S Güven Yılmaz S Koska İ Berdeli A Mir S. Evaluation of Development of Subclinical Atherosclerosis in Children With Uveitis. Clin Rheumatol (2018) 37(5):1305–8. doi: 10.1007/s10067-017-3741-3 28695435

[36] Martin TM Rosenbaum JT. An Update on the Genetics of HLA B27-Associated Acute Anterior Uveitis. Ocular Immunol Inflamm (2011) 19(2):108–14. doi: 10.3109/09273948.2011.559302 PMC3083239 21428748

[37] DeLay ML Turner MJ Klenk EI Smith JA Sowders DP Colbert RA. HLA-B27 Misfolding and the Unfolded Protein Response Augment Interleukin-23 Production and are Associated With Th17 Activation in Transgenic Rats. Arthritis Rheumat (2009) 60(9):2633–43. doi: 10.1002/art.24763 PMC2893331 19714651

[38] Zhong Z Su G Kijlstra A Yang P. Activation of the Interleukin-23/Interleukin-17 Signalling Pathway in Autoinflammatory and Autoimmune Uveitis. Prog Retin Eye Res (2021) 80:100866. doi: 10.1016/j.preteyeres.2020.100866 32422390

[39] Wakefield D Yates W Amjadi S McCluskey P. HLA-B27 Anterior Uveitis: Immunology and Immunopathology. Ocular Immunol Inflamm (2016) 24(4):450–9. doi: 10.3109/09273948.2016.1158283 27245590

[40] Pejnovic N Vratimos A Lee SH Popadic D Takeda K Akira S. Increased Atherosclerotic Lesions and Th17 in Interleukin-18 Deficient Apolipoprotein E-Knockout Mice Fed High-Fat Diet. Mol Immunol (2009) 47(1):37–45. doi: 10.1016/j.molimm.2008.12.032 19201478

[41] Cheng X Yu X Ding YJ Fu QQ Xie JJ Tang TT. The Th17/Treg Imbalance in Patients With Acute Coronary Syndrome. Clin Immunol (Orlando Fla) (2008) 127(1):89–97. doi: 10.1016/j.clim.2008.01.009 18294918

[42] Smith E Prasad KM Butcher M Dobrian A Kolls JK Ley K. Blockade of Interleukin-17A Results in Reduced Atherosclerosis in Apolipoprotein E-Deficient Mice. Circulation (2010) 121(15):1746–55. doi: 10.1161/CIRCULATIONAHA.109.924886 PMC2929562 20368519

[43] Erbel C Chen L Bea F Wangler S Celik S Lasitschka F. Inhibition of IL-17A Attenuates Atherosclerotic Lesion Development in apoE-Deficient Mice. J Immunol (Baltimore Md: 1950) (2009) 183(12):8167–75. doi: 10.4049/jimmunol.0901126 20007582

[44] Bayen H Bayen MC De Curzon HP Espinasse-Berrod MA Manderieux N Furia M. Involvement of the Posterior Eye Segment in HLA B27(+) Iridocyclitis. Incidence. Value of Surgical Treatment. J Francais D’ophtalmol (1988) 11(8-9):561–6. 3068282

[45] Rodriguez A Akova YA Pedroza-Seres M Foster CS. Posterior Segment Ocular Manifestations in Patients With HLA-B27-Associated Uveitis. Ophthalmology (1994) 101(7):1267–74. doi: 10.1016/S0161-6420(94)31179-1 8035991

[46] Kim SJ Chung H Yu HG. Posterior Segment Involvement in Korean Patients With HLA-B27-Associated Uveitis. Ocular Immunol Inflamm (2009) 17(1):26–32. doi: 10.1080/09273940802553261 19294570

[47] Dodds EM Lowder CY Meisler DM. Posterior Segment Inflammation in HLA-B27+ Acute Anterior Uveitis: Clinical Characteristics. Ocular Immunol Inflamm (1999) 7(2):85–92. doi: 10.1076/ocii.7.2.85.4015 10420203

