# Supplementary material for: Uveitis as a Risk Factor for Developing Acute Myocardial Infarction in Ankylosing Spondylitis: A National Population-Based Longitudinal Cohort Study
Source: Front Immunol. 2022 Jan 11;12:811664. doi: 10.3389/fimmu.2021.811664 (PMC8787117; doi:10.3389/fimmu.2021.811664)
Supplement: Supplementary file 1 [file Table_1.docx]

| **Table S1. Abbreviation, ICD-9-CM, and definition** | | |
| --- | --- | --- |
|  | **Abbreviation** | **ICD-9-CM / Definition** |
| **Study population:** |  |  |
| Ankylosing spondylitis | AS | 720.0; Outpatient visits≧ 3 or inpatient |
| Uveitis |  |  |
| Anterior uveitis |  | 364.0-364.3 |
| Posterior segment involvement |  | 363.0, 363.00-363.01, 363.03-363.08, 363.1, 363.10-363.12, 362.18, 363.20-363.21 |
| **Events:** Acute myocardial infarction | AMI | 410 |
| **Comorbidities:** |  |  |
| Diabetes mellitus | DM | 250 |
| Hyperlipidemia |  | 272 |
| Hypertension | HTN | 401-405 |
| Cerebrovascular accident | CVA | 430-438 |
| Congestive heart failure | CHF | 428 |
| Chronic obstructive pulmonary disease | COPD | 490-492, 494, 496 |
| Asthma |  | 493 |
| Coronary artery disease | CAD | 413-414 |
| Cardiomegaly |  | 429.3 |
| Metabolic syndrome | MetS | 277.7 |
| **Charlson comorbidity index revised** | CCI_R | CCI removed AMI, DM, hyperlipidemia, HTN, CVA, CHF, COPD, Asthma, CAD, cardiomegaly and MetS |
